# Ways to address health financing pressures and donor transition, Kenya

**DOI:** 10.2471/BLT.26.297090

**Published:** 2026-09-01

**Authors:** Colette SA Wesonga, Benard W Kulohoma

**Affiliations:** a Ortholog, PO Box 35723, City Square, 00200 Nairobi, Kenya.

For the last two decades, the national response to human immunodeficiency virus (HIV), tuberculosis and malaria in Kenya has been supplemented by external funding, including from the United States President’s Emergency Plan for AIDS Relief (PEPFAR), the Global Fund to Fight AIDS, Tuberculosis and Malaria, and other international initiatives. Some of these funds were awarded to build domestic vaccine and pharmaceutical manufacturing capacity. While these donor funds previously operated along independent cycles and strategic trajectories, between 2025 and 2030 these external mechanisms are expected to taper simultaneously, creating a funding transition challenge. Kenya therefore faces a crucial conjuncture of absorbing substantial financing gaps through domestic resource mobilization while continuing to develop local production capacity.^1^^,^^2^ Furthermore, epidemiological data indicate that new HIV, tuberculosis and malaria infections in Kenya remain a consistent challenge to Kenya’s health-care system, emphasizing the need for sustained control efforts to achieve health-related global targets.^3^^–^^5^ The expected decrease in international funding serves as a resilience evaluation for the national health system.

The United States executive order issued in January 2025, which initiated a 90-day review and suspension of United States foreign assistance for global health, had immediate operational effects on community health monitoring, clinics and health workers embedded within many local systems, including those in Kenya, highlighting a critical gap in domestic health financing absorption capacity during acute resource deficits.^1^^,^^6^ The transition of the United States Department of State away from existing global procurement and supply chain mechanisms for HIV, tuberculosis and malaria commodities by mid-2026 threatens continuous product availability across clinical facilities unless domestic procurement pipelines are fully operational. In 2025, the United States introduced the America First Global Health Strategy, which aims to align foreign health assistance with national interests, focus on mutual investments and bilateral agreements, and enable long-term self-reliance. Under this strategy, PEPFAR negotiated five-year bilateral memoranda of understanding (2026–2030) with recipient countries. In Kenya, these bilateral negotiations aimed to transition full responsibility for HIV programmes, personnel and commodity financing to national authorities by 2028.

In the current challenging global context, the Global Fund is also conducting a strategic shift towards advancing recipient country domestic self-reliance. Globally, the Global Fund replenishment for 2026–2028 secured 12.64 billion United States dollars (US$) against an estimated US$ 18 billion target,^7^ necessitating reprogramming reductions to accommodate this shortfall. Kenya’s Global Fund Grant Cycle 7 allocation (July 2024–June 2027) was reduced from an initial approval of US$ 408 million to US$ 354 million, while the subsequent Global Grant Cycle 8 HIV allocation faces a reduction of US$ 45.61 million (about 18%), and parallel funding reductions are projected for malaria and tuberculosis.^8^ With the reduction of global financing and needed increase of countries’ contributions, rigorous cofinancing requirements ensure commitment in domestic resource mobilization. In Kenya, this cofinancing with the Global Fund faces the additional challenge of external debt servicing deadlines that leave little room to make health investments.

To mitigate long-term external dependency, international actors, including the World Bank, International Finance Corporation and Gavi, the Vaccine Alliance, have supported local manufacturing initiatives. However, pharmaceutical technology transfer and facility validation typically require 5 to 10 years, whereas the anticipated withdrawal of external support converges around 2028–2030. Consequently, domestic manufacturing capacity is being established concurrently with, rather than ahead of, donor phase-outs.

The simultaneous dwindling of international funding in the coming years requires strategic alignment. To safeguard public health gains and ensure uninterrupted service delivery, we suggest that policy-makers and health authorities should prioritize four actions. First, institutionalize domestic financing transition strategies by establishing multiyear treasury allocations to absorb the recurrent costs for health workers and commodities (such as medicines, diagnostics and supplies) currently dependent on external grants.^2^ Second, protect primary health-care services and those receiving them by safeguarding targeted prevention programmes for high-risk and key populations, ensuring that fiscal contractions do not disproportionately affect marginalized groups.^5^ Third, strengthen supply chain resilience by accelerating domestic procurement mechanisms and regional manufacturing partnerships to prevent commodity stock-outs during international procurement transitions.^9^ Fourth, strengthen data systems and surveillance by restoring and maintaining robust monitoring and evaluation infrastructure to accurately measure health outcomes and track service retention in real time.^10^ Without adequate financing of the health system when international funding declines, decades of hard-won public health gains risk being reversed, leaving populations vulnerable to preventable disease and placing an increasing economic burden on health systems.
